# Research progress of artificial intelligence in high-throughput drug screening

**DOI:** 10.3389/fphar.2026.1847031

**Published:** 2026-06-04

**Authors:** Xiaoyong Liu, Xiaoli Ren, Pengxi Li, Xueguo Li, Xiaoping Ren, Chaoya Sui, Hailun Zhou, Fen Luo, Ling Tao

**Affiliations:** 1 Chongqing Chemical Industry Vocational College, Chongqing, China; 2 Department of Oncology, The People’s Hospital of Chongqing Liang Jiang New Area, Chongqing, China

**Keywords:** artificial intelligence, data analysis, deep learning, high-throughput drug screening, image recognition, virtual screening

## Abstract

High-throughput screening (HTS) is widely used in modern drug discovery. It enables batch activity testing of compounds and provides important support for the identification of active compounds. However, its screening efficiency and accuracy need to be improved. To address this issue, artificial intelligence (AI) has been gradually integrated into the HTS workflow. Leveraging the advantages of machine learning (ML) and deep learning (DL), AI optimizes applications in structure-based and ligand-based virtual screening, combination drug screening, image analysis, and post-screening data analysis and interpretation, driving the intelligent development of drug discovery. This paper reviews recent research progress in the application of AI in HTS, discusses the implementation of machine learning models, and summarizes key AI applications in HTS-related compound screening, image recognition, and hit identification from complex screening data, aiming to accelerate the development of innovative drugs.

## 1 Introduction

High-throughput screening (HTS), as a critical step in drug discovery, is a key tool in modern drug development. Through parallel experimental design and automated operation, it accelerates the discovery and optimization of novel drug candidate molecules (Jiang et al., 2025). However, in the face of the increasingly large compound libraries and complex biological target networks, traditional HTS techniques face severe challenges: they suffer from high false-positive/false-negative rates due to interference from biological noise, rely heavily on predefined rules and prior knowledge for hit selection, and struggle to fully exploit the massive multi-dimensional data generated during screening (e.g., multi-parameter readouts, time-series imaging, and omics data).

The rapid development of artificial intelligence (AI), particularly machine learning (ML) and deep learning (DL), provides powerful tools to overcome these limitations. ML algorithms (e.g., random forest, support vector machines) excel at learning patterns from labeled experimental data, enabling accurate prediction of compound activity and toxicity without explicit mechanistic rules. DL, a subfield of ML, uses multi-layer neural networks to automatically extract hierarchical features from raw data (e.g., images, molecular graphs) and handle non-linear relationships that traditional methods cannot capture. By integrating ML/DL, HTS workflows can achieve adaptive hit selection, reduction of noise-induced errors, and efficient mining of subtle signals in large-scale datasets. By leveraging the algorithmic advantages of ML and DL, AI exhibits great potential in structure-based and ligand-based virtual screening, combination drug screening, high-content screening (HCS) image analysis, and large-scale data processing (Myung et al., 2024). Furthermore, with the Turing Award being repeatedly awarded to pioneers in the AI field and the breakthrough of AlphaFold in protein structure prediction, the application of AI in biomedicine has become increasingly extensive and important. These advances not only highlight the prospective application of AI in drug discovery but also drive the leapfrog development of HTS technology.

At present, the deep integration of AI and HTS has not only significantly improved screening efficiency but also greatly accelerated the progress of drug development. With continuous optimization of algorithms and continuous upgrading of computing resources, the application prospect of AI in HTS is becoming broader. This paper provides a concise overview of recent research progress in the application of AI in the field of HTS and analyzes its performance in different task scenarios in depth with practical cases.

**FIGURE 1:**
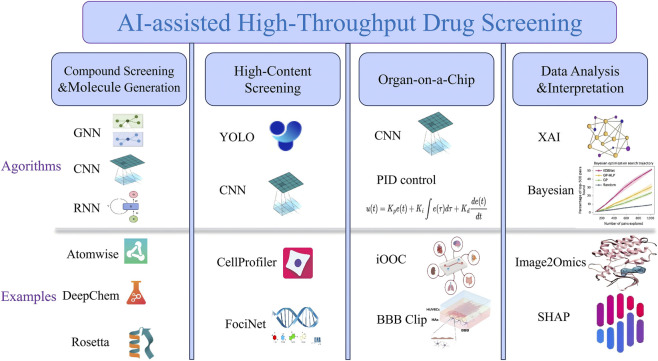
Applications and algorithms of AI-assisted high-throughput drug screening. GNN: Graph neural network; CNN: Convolutional neural network; RNN: Recurrent neural network; XAI: Explainable artificial intelligence.

Figure 1 illustrates several key applications of AI in HTS, including compound screening, molecule generation, high-content screening, organ-on-a-chip, Data Analysis and Interpretation. Representative AI-driven platforms/examples include Atomwise (deep learning-based virtual screening for target-directed libraries), Rosetta (macromolecular modeling and docking for structure-based design), and DeepChem (graph neural network for pharmacokinetic prediction).

## 2 Compound screening

**FIGURE 2:**
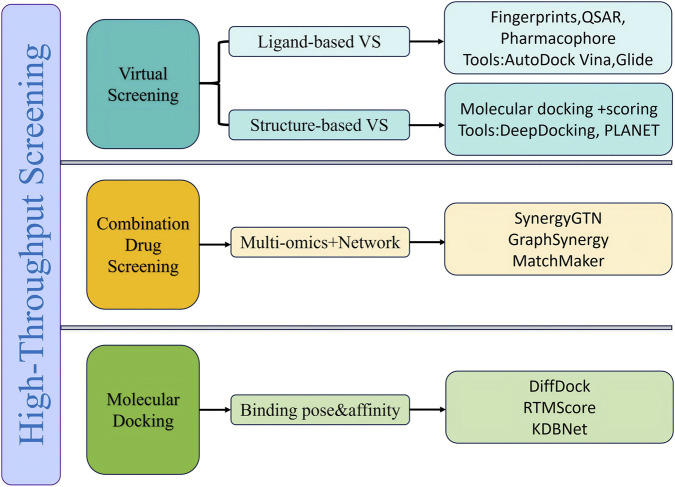
Overview of AI-assisted compound screening strategies in HTS.

Compound screening is a critical step in the drug discovery pipeline, aiming to identify promising active compounds from large-scale chemical libraries. The introduction of artificial intelligence (AI) has significantly improved screening efficiency, accuracy, and the speed of drug discovery (Li et al., 2024). To provide a clear overview of the AI-empowered strategies discussed in this section, Figure 2 summarizes the definitions, purposes, and representative software tools for virtual screening, combination drug screening, and molecular docking.

### 2.1 Virtual screening

Virtual screening (VS) is a computational technique to rapidly evaluate large compound libraries and prioritize candidates for experimental testing. VS is broadly classified into two categories: ligand-based virtual screening (LBVS) and structure-based virtual screening (SBVS). LBVS works when the 3D structure of the target is unknown, leveraging known active ligands; SBVS requires the 3D structure of the target and predicts binding via docking or other physical models.

#### 2.1.1 Ligand-based virtual screening (LBVS)

Ligand-based virtual screening (LBVS) is a computational method that screens compound libraries to identify potential drug molecules with strong binding affinity to known target proteins (Liu et al., 2021). LBVS is a versatile computational strategy independent of the 3D structures of target proteins, covering a variety of core strategies including mining structure-activity relationships (SAR) and quantitative structure-activity relationships (QSAR) from known active ligands, comparing molecular fingerprints, physicochemical properties, and pharmacophore models across compound libraries, identifying molecules with similar biological activity, binding modes or functional mechanisms to known active compounds, and prioritizing candidate compounds for experimental verification in early hit discovery and lead optimization. LBVS is widely used in rapid hit identification, scaffold hopping, selectivity optimization, and phenotypic activity prediction especially when the 3D structures of target proteins are unknown or poorly resolved (Liu et al., 2021; Dara et al., 2022).

Chronologically, the application of AI in LBVS began with traditional ML algorithms, which were then extended and largely superseded by deep learning methods.

Traditional ML algorithms such as support vector machine (SVM), random forest (RF), and K-nearest neighbors (KNN) have been widely used in LBVS. These methods construct QSAR models using chemical descriptors (e.g., molecular fingerprints, topological indices) and effectively predict ligand-target binding affinity (Orsi et al., 2024). They address some HTS limitations by reducing false positives through supervised classification and enabling virtual screening of millions of compounds *in silico*, thus saving time and cost. For small-to medium-sized datasets, these algorithms are efficient and their classification/regression capabilities can rapidly identify potential active ligands. Representative successful studies based on traditional ML algorithms include: RF models built on molecular descriptors and fingerprints successfully identified novel adenosine A2A receptor antagonists with micromolar activity and validated 8 hits from 100 selected compounds (Goßen et al., 2023); SVM achieved over 80% accuracy in screening JAK2 inhibitors from a library of 100,000 compounds and obtained 3 nanomolar-level verified hits (Orsi et al., 2024); KNN models using topological descriptors accelerated the screening of ruthenium complexes and discovered two new antibacterial agents against drug-resistant *Staphylococcus aureus* (Orsi et al., 2024); Naïve Bayes and Logistic Regression were combined to pre-filter toxic compounds, reducing experimental attrition by approximately 40% in the discovery of anti-inflammatory lead compounds (Dara et al., 2022). However, traditional ML algorithms still rely heavily on manual feature engineering, cannot automatically capture high-order non-linear interactions, and lack scalability for ultra-large (billion-scale) datasets.

In contrast, deep learning (DL) is a subfield of ML that uses hierarchically connected nonlinear processing units to perform feature extraction and transformation, construct multi-level feature representations, and learn abstract concepts. Compared with traditional ML methods, DL shows stronger advantages in non-linear modeling and automatic feature extraction, especially for high-dimensional and complex data. DL includes various neural network architectures, such as convolutional neural network (CNN), recurrent neural network (RNN), and generative adversarial network (GAN). These models can automatically extract high-level features from ligand-target binding processes, thereby significantly improving the accuracy and generalization ability of virtual screening (Du et al., 2023).

In recent years, DL models based on graph neural networks (GNN) have been widely adopted in LBVS. The DeepChem platform uses GNN models to predict the pharmacokinetic and toxicity profiles of compounds. In comparative tests, the platform substantially improved prediction performance across multiple key endpoints. In metabolic stability prediction, the Top-50 accuracy of the DeepChem model exceeded the maximum accuracy (69.5%) of the baseline rule-based system, demonstrating excellent performance in candidate compound identification. With the assistance of AI models, researchers can eliminate compounds that fail to meet pharmacokinetic criteria at an early stage, reducing the time and cost of subsequent screening.

The Deep-PK platform also uses GNN to predict the pharmacokinetics and toxicity of small-molecule drugs. In a comparison with ADMETlab 2.0 across 53 prediction tasks, Deep-PK outperformed the baseline model in 46 tasks: the Matthews correlation coefficient (MCC) for the “NR-A” nuclear receptor activation endpoint increased by 0.43, and the average MCC for metabolism-related tasks rose by 0.105. Among 36 toxicity prediction tasks, Deep-PK showed improved performance for 30 endpoints, with the overall average MCC increasing from 0.58 to 0.71 (Myung et al., 2024).

The MD-GNN model inputs molecular structures into GNN and fuses experimental and computational data, improving molecular property prediction. When predicting logP for 10 modified molecules, the model successfully identified 9 potentially active compounds. Comparative experiments showed that after feature fusion, MD-GNN reduced the mean absolute error on the test set by approximately 0.2–0.3 compared with using GNN or molecular descriptors alone, indicating that feature fusion significantly enhanced prediction stability and accuracy. Moreover, compared with classic GNN algorithms, MD-GNN reduced the average error by 0.2 on the test set, demonstrating its advantages in multi-endpoint prediction (Chen et al., 2023).

The FP-GNN model combines molecular fingerprints with GNN and achieved an average area under the curve (AUC) of 0.849 on phenotypic screening datasets across 14 breast cancer and normal cell lines. It also outperformed traditional molecular fingerprint models and classic GNN models in noise resistance and interpretability (Cai et al., 2022).

Ligandformer is another advanced AI model for compound property prediction. It extracts local and global features from compound structures via multi-layer self-attention mechanisms and fuses attention maps from different network modules, achieving robust and interpretable predictions. On water solubility, Caco-2 permeability, and Ames mutagenicity, Ligandformer outperformed recently developed MPNN and SAMPN models, with AUC values of 0.98, 0.89, and 0.92, respectively. Furthermore, visualization of attention coefficients allows researchers to intuitively identify atoms or fragments that contribute most to property prediction, providing guidance for compound structure optimization.

The successful application of these models provides new perspectives for drug discovery and accelerates the screening of drug candidate molecules. By learning large amounts of compound and target data, AI techniques automatically extract complex relationships between molecular structures and biological activities. These methods not only optimize ligand-target interaction prediction but also discover potential active compounds in massive datasets, identifying weak connections difficult to detect using traditional approaches. In addition, AI can guide molecular generation and drug design, helping to discover novel compound structures or optimize existing drugs. The integration of AI and LBVS not only accelerates drug discovery but also provides strong support for rational drug design and personalized therapy. For instance, GNN-enhanced LBVS guided the structural optimization of 5-HT1F receptor agonists and improved their oral bioavailability and central nervous system penetration for migraine treatment (Liu et al., 2021); ML-based LBVS stratified patients according to tumor mutation profiles and prioritized patient-specific EGFR inhibitors, leading to improved response rates in clinical cohorts (Li et al., 2024); deep learning-based LBVS identified SARS-CoV-2 3CLpro inhibitors with optimized binding affinity and metabolic stability, advancing two candidates to *in vivo* tests (Du et al., 2023); AI-assisted LBVS also designed patient-tailored azole derivatives with reduced host toxicity for personalized antifungal therapy (Chen et al., 2023).

However, the performance of these methods heavily depends on dataset quality and evaluation strategies. Studies have shown that inappropriate data splitting can lead to data leakage. For instance, highly similar compound conformations or shared binding pockets between training and test sets can artificially inflate prediction accuracy (Tran-Nguyen et al., 2020). Furthermore, in molecular docking and LBVS, if conformation or pocket information leakage is not strictly controlled, models may learn structural priors rather than genuine binding rules. The choice of cross-validation strategy also significantly affects model generalization (Moshawih et al., 2024). Therefore, future research should adopt more rigorous dataset construction and validation protocols to ensure reliable model evaluation and clinical translatability.

#### 2.1.2 Structure-based virtual screening (SBVS)

Structure-based virtual screening (SBVS) relies on the three-dimensional structure of the biological target (e.g., protein, nucleic acid) to predict how small molecules bind to its active site. Traditional SBVS uses molecular docking to generate putative binding poses and scoring functions to rank compounds. While effective, conventional scoring functions often suffer from inaccurate energy calculations, neglect of protein flexibility, and poor correlation with experimentally measured affinities.

AI has significantly advanced SBVS by replacing or augmenting classical scoring functions with ML/DL models that learn from protein-ligand complex data. These AI-driven approaches can account for non-additive interactions, solvation effects, and induced fit in a data-driven manner, thereby improving hit enrichment compared to traditional docking. For example, the DL docking platform DeepDocking, when combined with the FRED docking program, can rapidly and accurately compute docking scores of 1.36 billion molecules from the ZINC 15 library against 12 major target proteins, demonstrating up to 100-fold data reduction and 6000-fold enrichment of high-scoring molecules (Gentile et al., 2020). The DL algorithm KDBNet integrates 3D protein and molecular structure data to predict binding affinity. Experiments show that it outperforms existing DL models in predicting kinase-drug affinities. When integrated with a Bayesian optimization framework, KDBNet enables data-efficient active learning and accelerates the exploration and utilization of various high-binding kinase-drug pairs (Luo et al., 2023). Studies have also reported that active learning and Bayesian optimization are effective approaches for chemical space search and key to improving screening efficiency (Cao et al., 2024). Uni-Mol, a universal 3D molecular representation learning framework, significantly expanded molecular representation capabilities and application scopes. In protein-ligand binding conformation prediction, its predicted root-mean-square deviation can be as low as 1.23 Å, and in molecular conformation generation, the success rate exceeds 90%. DiffDock combines DL with graph convolutional networks to perform blind protein-ligand docking, significantly improving docking accuracy and computational speed (Fortela et al., 2024). RTMScore is a novel scoring function that builds a residue-atom distance likelihood model using a mixture density network. In docking tests on the CASF benchmark, RTMScore achieved Top-1 docking success rates of 93.1% and 89.7%, significantly outperforming most DL and traditional methods. The PLANET model integrates 3D structures of protein binding pockets and 2D ligand structures. Evaluated on major public datasets, PLANET achieved accuracy comparable to the traditional docking program Glide, but with computation time less than 1% of Glide’s (Zhang S. et al., 2023). These AI-enhanced SBVS tools effectively expand the chemical space that can be explored and reduce the need for exhaustive brute-force docking.

### 2.2 Combination drug screening

Combination drug screening is a strategy based on drug combinations that enhances efficacy, overcomes drug resistance, or reduces adverse effects by combining drugs with different mechanisms of action. The application of AI in combination drug screening helps identify effective drug pairs.

SynergyGTN uses GNN to predict synergistic drug combinations by integrating drug graph features and cell line gene expression profiles. In five-fold cross-validation, it achieved AUC-ROC and AUC-PR values of 0.98 and 0.97, respectively, with an accuracy of 0.93, representing a 5%–8% improvement over the current state-of-the-art methods. Validation on an independent ASTRAZENECA dataset showed a 13% increase in balanced accuracy, demonstrating high accuracy and strong generalization in drug combination screening (Alam et al., 2024).

The GraphSynergy model is based on protein-protein interaction networks and uses graph convolutional networks and attention mechanisms to predict synergistic anticancer drug combinations. The model achieved accuracies of 0.7553 and 0.7557 on the DrugCombDB and Oncology-Screen datasets, respectively, representing improvements of approximately 12% and 11% over the recently published DeepSynergy algorithm. Key proteins assigned high weights during training play important roles in molecular functions and biological processes such as transcription and transcriptional regulation, thereby improving the accuracy of drug combination prediction (Yang et al., 2021).

SynergyX is a multimodal interaction network that integrates drug and cell line multi-omics data to achieve high-precision prediction of antitumor drug combinations, outperforming the second-best model by 2%–10%. Cross-dataset validation confirmed its robustness, with only a∼2% drop in R^2^ value. It can capture drug-cell interactions via a cross-modal fusion encoder, ensuring prediction interpretability (Guo et al., 2024).

The MatchMaker model combines drug chemical structures and cell line gene expression profiles. It first learns embeddings for single drugs in specific cell lines and then concatenates embedding vectors to predict synergistic effects, enabling quantification of drug interactions. Compared with high-throughput screening, this model drastically reduces experimental requirements. In comparative experiments, MatchMaker outperformed traditional ML methods including DeepSynergy, TreeCombo, random forest, and ElasticNet, demonstrating its advantages in synergistic drug combination prediction (Kuru et al., 2022).

With the emergence of these methods, research focus has gradually shifted from purely pursuing predictive performance toward clinical applicability. On the one hand, mechanistic interpretability has become an important consideration. Many models use attention weights, graph pooling, or feature attribution methods to locate genes or pathways critical for drug synergy, providing biological explanations for predictions. On the other hand, balancing efficacy and toxicity has gained increasing attention. Some studies simultaneously incorporate phenotypic data related to efficacy and toxicity into models, or use multi-task learning frameworks to predict synergy and potential adverse reactions in parallel, thereby avoiding recommending highly effective but highly toxic combinations (Monem et al., 2024). Furthermore, multi-objective optimization strategies have been introduced into combination drug screening, allowing models to find trade-off solutions between maximizing efficacy and minimizing toxicity and output more clinically feasible candidate regimens (Gevertz and Kareva, 2023).

AI not only significantly improves the efficiency and accuracy of combination drug screening but also drives the field toward greater clinical translational potential by enhancing interpretability, introducing efficacy-toxicity trade-offs, and applying multi-objective optimization.

### 2.3 Molecular docking

Molecular docking is one of the important strategies for screening potential active compounds and supporting subsequent lead discovery. In recent years, AI-driven receptor-ligand docking models have advanced rapidly. Such models can not only directly generate complex 3D atomic coordinates using equivariant neural networks (Qiao et al., 2024) but also learn the probability density distribution of distances between receptors and ligands, enabling efficient prediction of binding conformations (Méndez-Lucio et al., 2021).

Molecular docking mainly involves analyzing binding site features of target proteins and their interactions with small-molecule drugs based on 3D protein structures, using affinity scoring functions to evaluate protein-drug binding affinity, and selecting candidate compounds with high predicted scores from large compound libraries for subsequent bioactivity testing.

For example, the DL docking platform DeepDocking, when combined with the FRED docking program, can rapidly and accurately compute docking scores of 1.36 billion molecules from the ZINC 15 library against 12 major target proteins, demonstrating up to 100-fold data reduction and 6000-fold enrichment of high-scoring molecules (Gentile et al., 2020).

The DL algorithm KDBNet integrates 3D protein and molecular structure data to predict binding affinity. Experiments show that it outperforms existing DL models in predicting kinase-drug affinities. When integrated with a Bayesian optimization framework, KDBNet enables data-efficient active learning and accelerates the exploration and utilization of various high-binding kinase-drug pairs (Luo et al., 2023). Studies have also reported that active learning and Bayesian optimization are effective approaches for chemical space search and key to improving screening efficiency (Cao et al., 2024).

Early successes of ML in affinity prediction have stimulated interest in DL models, which may outperform traditional scoring functions in processing 3D structural and non-structural data. For example, Uni-Mol, a universal 3D molecular representation learning framework, significantly expanded molecular representation capabilities and application scopes. In protein-ligand binding conformation prediction, its predicted root-mean-square deviation can be as low as 1.23 Å, and in molecular conformation generation, the success rate exceeds 90%.

Besides GNN-based methods, DiffDock combines DL with graph convolutional networks to perform blind protein-ligand docking, significantly improving docking accuracy and computational speed. This method exhibited high efficiency in evaluating the binding affinity of per- and polyfluoroalkyl substances to blood proteins, providing a rapid tool for studying their fate in humans and potential molecular mechanisms (Fortela et al., 2024).

RTMScore is a novel scoring function that builds a residue-atom distance likelihood model using a mixture density network. In docking tests on the CASF benchmark, RTMScore achieved Top-1 docking success rates of 93.1% and 89.7%, significantly outperforming most DL and traditional methods. Moreover, in forward screening tasks, RTMScore identified 66.7%, 83.6%, and 90.1% of the highest-affinity ligands among the top 1%, 5%, and 10% of candidate molecules, respectively, and maintained clear advantages in reverse screening. RTMScore exhibited superior overall performance compared with mainstream existing methods in both docking accuracy and screening power.

The PLANET model integrates 3D structures of protein binding pockets and 2D ligand structures. Evaluated on major public datasets, PLANET achieved accuracy comparable to the traditional docking program Glide, but with computation time less than 1% of Glide’s (Zhang Y. et al., 2023).

These AI-enhanced molecular docking algorithms effectively improved performance and hold promising application prospects.

### 2.4 Molecular generation and large model-driven design

With the rapid development of generative AI, molecular generation and optimization have become important complementary approaches to drug screening. Unlike traditional ligand- or structure-based virtual screening, these methods can directly generate novel molecules with specific properties in chemical space, enabling an integrated “design-screen” workflow and accelerating the discovery of active compounds.

The rise of diffusion models has significantly advanced molecular generation. Compared with early methods such as variational autoencoders and generative adversarial networks, diffusion models exhibit greater stability and expressive power when modeling complex molecular distributions. For example, SE (3)-equivariant diffusion models preserve the invariance of 3D molecular structures under rotation and translation, achieving excellent performance in 3D generation of protein-ligand complexes and flexible docking (Zhu et al., 2024). Furthermore, cross-scale protein-ligand modeling can directly generate small-molecule structures matching target binding pockets. By introducing multi-objective optimization that simultaneously considers synthetic accessibility and docking scores, the model’s synthetic accessibility score can be improved to 0.78, showing clear advantages over traditional and non-optimized models (Cremer et al., 2024).

Large language models have also been introduced to model molecular sequences and graph structures. 3D-MolT5 unifies the encoding of 1D sequences, 3D structures, and textual information of molecules, enabling cross-modal understanding. In downstream tasks including molecular property prediction, 3D molecular description generation, and text-driven molecule generation, 3D-MolT5 outperformed traditional baseline models.

ChemLLM further extends multimodal capabilities to molecular image processing, simultaneously performing molecular image captioning, image property prediction, image-to-SMILES translation, and multi-objective molecular image design. It outperformed existing multimodal large models in these tasks while maintaining strong task generality.

In addition, recently proposed multimodal chemical and biological foundation models can simultaneously process molecular structures and protein sequences, and even integrate molecular images to enable cross-scale interaction prediction (Luo et al., 2024). Such methods not only improve the accuracy of molecular screening and binding prediction but also show potential in complex tasks such as antibody-antigen interactions and protein-small molecule co-design.

Typical compounds successfully discovered by these generative methods include: a novel JAK1 ligand generated by an SE (3) diffusion model with an IC_50_ value of 47 nM and 12-fold higher selectivity over the parent lead, which has been verified in cell assays (Zhu et al., 2024); CDK4/6-targeted PROTACs generated by 3D-MolT5 showing significant anti-tumor activity in breast cancer organoids (Luo et al., 2024); new Gram-negative antibacterial macrocycles designed by ChemLLM overcoming efflux pump resistance and validated in animal infection models (Cremer et al., 2024); a direct KRAS G12D binder (Kd = 230 nM) generated by the Pilot diffusion model targeting the previously undruggable KRAS target (Cremer et al., 2024); selective DDR1 inhibitors for idiopathic pulmonary fibrosis generated by Uni-Mol, with one candidate entering preclinical development (Qiao et al., 2024).

## 3 Image recognition and data analysis

The combination of image recognition technology and deep learning (DL) algorithms enables efficient processing of large-scale experimental data and promotes the automation of high-throughput screening (HTS). Currently, it has been widely applied in high-content screening (HCS), organoids, and organ-on-a-chip technologies. Beyond image analysis, AI has emerged as a critical tool for downstream data analysis and interpretation-transforming raw screening outputs into biological insights and actionable hits.

### 3.1 High-content screening

Since Cellomics launched the first high-content imaging platform for HTS in 1997, HCS has become one of the core technologies in drug discovery. HCS is an HTS technique based on cellular image analysis. The integration of DL and multi-omics data analysis has significantly improved the efficiency of cell phenotype classification and the accuracy of prediction. Traditional HCS methods mostly use two-dimensional cell culture and proprietary algorithms for image analysis. However, with the development of open-source software such as CellProfiler, Definiens, Advanced Cell Classifier, and DL technology, image analysis has expanded from simple quantitative measurements to in-depth analysis of complex cellular phenotypes. Through image segmentation and feature extraction algorithms, these software tools can extract cellular morphological features from complex images, providing strong support for drug discovery (Way et al., 2023).

By combining HCS with multi-omics data including genomics and proteomics, researchers can gain a more comprehensive understanding of cellular responses to drugs and their mechanisms of action, thereby accelerating the discovery of new therapies. Using the Image2Omics DL approach, researchers successfully predicted the abundance of several transcripts and proteins in M1 and M2 macrophages from high-content images, demonstrating that cell imaging may serve as a scalable alternative to multi-omics measurements. In addition, DL-based detection using YOLOv5 exhibited excellent performance in macrophage HCS, significantly improving detection accuracy in high-cell-density regions and increasing processing speed by approximately twofold (Ong et al., 2023). A cross-domain consistency learning method has been proposed to address batch effects. Without high-quality labels, it eliminates batch-specific noise, improves the classification accuracy of cell phenotypes, and further enhances the ability to identify therapeutic effects. HCS provides important support for phenomics, helps reveal potential cellular changes during treatment, and offers new directions for drug screening.

Some researchers constructed a DL-based morphological screening platform using LPS-stimulated mouse macrophages. Cellular phenotypes were labeled with seven fluorescent probes, and a deep neural network was trained to distinguish between normal and inflammatory states. From 2259 compounds with known mechanisms, 12 active compounds that significantly regulate LPS-induced cytokine expression were screened, among which 7 were MEK1/2-specific inhibitors. This high-content analysis method provides a new strategy for anti-inflammatory drug discovery (Lau et al., 2024). Furthermore, the DL platform FociNet can automatically segment whole-field fluorescence images and analyze DNA damage in individual cells. After training on 6000 annotated mononuclear images, FociNet satisfactorily classifies cells into normal, damaged, or signal-negative states. It was also applied to analyze more than 5000 focus images from HCS of 315 natural compounds from traditional Chinese medicine, successfully identifying several novel active compounds (such as ifosfamide) that significantly reduce DNA damage markers, among which isoliquiritigenin can notably decrease double-strand breaks. This study provides a new approach for AI-assisted drug discovery based on microscopic evaluation of DNA damage (Chen et al., 2020).

In HCS, data quality is critical for model performance. A typical end-to-end pipeline includes raw image acquisition, annotation strategies, preprocessing, and feature extraction. Annotation strategies can adopt manual annotation, semi-supervised learning, or automatic labeling based on known phenotypes to ensure the accuracy of training data. Batch effects are common in high-throughput experiments; systematic biases may be introduced by different experimental batches, imaging conditions, or staining batches, which can be corrected using batch normalization, adversarial training, or cross-domain consistency learning (Arevalo et al., 2024).

### 3.2 Organoids

Organoids are three-dimensional (3D) culture systems derived from stem cells or tissue-specific progenitor cells that can mimic the complex structure and function of organs *in vitro*. Therefore, they are widely used in disease modeling, personalized medicine, and drug screening. However, traditional organoid analysis methods usually rely on manual evaluation or inefficient computational approaches, which struggle to meet the demands of HTS. The rapid development of DL technology in fields such as image segmentation, object detection, and spatiotemporal analysis has shown great potential in organoid research. AI based on DL provides powerful support for automated detection, morphological analysis, functional evaluation, and drug screening of organoids.

Morphological features such as size, shape, and structural integrity of organoids are important indicators for evaluating their viability, proliferation, and drug response. Nevertheless, the 3D culture environment of organoids makes imaging susceptible to focal plane, light scattering, and sample heterogeneity, increasing the difficulty of morphological analysis. In recent years, DL has shown outstanding performance in organoid morphological analysis. For example, OrganelX and OrgaSegment employ DL-based segmentation methods to achieve automatic detection and morphological quantification of mouse liver organoids and cystic fibrosis organoids, improving measurement accuracy and consistency (Lefferts et al., 2024). In addition, Deep-Orga uses the lightweight neural network RepVGG to optimize computational efficiency, achieving mean average precision above 74% and 48% in medium and small organoid detection, respectively, providing a feasible solution for high-throughput analysis (Leng et al., 2024).

Besides morphological features, dynamic behaviors such as growth rate, differentiation tendency, and cell death of organoids are also important parameters for measuring biological function. Traditional ATP assays only provide overall growth viability information and cannot resolve the responses of individual organoids. AI-based video analysis methods, such as spatiotemporal models using SAM and DINOv2, can extract high-dimensional features from long-term time-lapse microscopy data and achieve accurate prediction of organoid drug responses, with an average prediction error of only 0.1755 across four validation sets (Fillioux et al., 2023). Furthermore, Cellos combines traditional computational methods with DL to distinguish different cell types in 3D organoids and quantitatively analyze their spatial distribution. In mixed fluorescence-labeled organoids, Cellos can accurately calculate the proportion of different clonal cells and track clonal-specific responses under drug treatment, further refining the functional evaluation of organoids.

With the widespread application of organoid models in HTS, efficiently analyzing their morphological and response characteristics has become a key issue. AI-based HTS strategies not only improve experimental efficiency but also optimize screening workflows. For example, 3D-Organoid-SwinNet integrates the Transformer model to achieve more accurate organoid segmentation, with a Dice score as high as 94.9% on the validation set. With only about 21 million parameters, it provides a more efficient analytical tool for protein readout and drug screening (Sohaib et al., 2025). In addition, ACU2Net improves the U-Net architecture and significantly enhances segmentation accuracy in bladder cancer organoid drug screening, with sensitivity, specificity, and F1-score reaching 94.81%, 88.50%, and 91.54%, respectively, offering a reliable tool for evaluating anticancer drug effects (Zhang Y. et al., 2023).

Key advances in drug discovery driven by AI-enabled organoid analysis include: high-throughput profiling of 120 clinical liver cancer organoids via Deep-Orga and OrganelX has identified a unique FGFR inhibitor sensitivity signature for patient stratification in Phase 2 clinical trials (Leng et al., 2024); OrgaSegment quantified CFTR-dependent fluid secretion in cystic fibrosis organoids and accelerated the approval of triple-combination CF therapies by predicting patient responses *ex vivo* (Lefferts et al., 2024); 3D-Organoid-SwinNet analyzed over 300 clinical colorectal cancer organoids and repurposed trifluridine for rare BRAF-mutant subtypes, which was validated in compassionate-use cohorts (Sohaib et al., 2025); ACU2Net accurately predicted the clinical response of bladder cancer organoids to anti-FGFR3 agents, reducing *in vivo* animal tests by approximately 70% and shortening lead optimization to 6 months (Zhang X. Y. et al., 2023).

### 3.3 Organ-on-a-chip

Organ-on-a-chip is a biomimetic *in vitro* system based on microfluidic cell culture. Compared with traditional two-dimensional cell culture or animal models, organ-on-a-chip exhibits higher physiological relevance and provides a dynamic experimental environment, more realistically simulating the physiological states and disease processes of human organs. HTS using organ-on-a-chip often relies on an end-to-end data processing pipeline, including image acquisition, preprocessing, segmentation and feature extraction, dynamic behavior analysis, and predictive model training, to ensure the systematic conversion of raw imaging data into functional conclusions.

Maintaining a stable and dynamic cell culture environment is a major challenge in the organ-on-a-chip field, especially in multiple organ system (MOS) models, where environmental stability and dynamic regulation are more difficult. Since the human body is a complex physiological system, MOS integrates multiple cell culture chambers through microchannels to simulate the overall physiological function under drug action, thereby more comprehensively analyzing dose-responses and toxicity mechanisms (Joseph et al., 2022). Therefore, ensuring the stability of organ-on-a-chip under dynamic culture conditions is crucial for simulating the real *in vivo* environment. AI technologies such as DL have been widely used to analyze multidimensional data from organ-on-a-chip sensors, which can monitor key physiological parameters including pH, oxygen levels, metabolic activity, and even cell morphology in real time to optimize experimental conditions, improve data accuracy, and enhance experimental reproducibility (Deng et al., 2023).

The intelligent organ-on-a-chip (iOOC) system can provide real-time feedback to dynamically regulate electrical, chemical, or mechanical stimuli to maintain the homeostasis of organ models, thereby more accurately simulating the feedback regulatory mechanisms in physiological systems. By drawing on closed-loop control strategies from engineering, iOOC can integrate biosensors to monitor key physiological parameters, compare measured data with target states, calculate errors, and adjust stimulus inputs. Common control methods include on/off control, proportional-integral-derivative (PID) control, and predictive optimization strategies combined with ML (Li Z. Y. et al., 2024). The real-time feedback loop between AI and organ-on-a-chip not only accelerates the discovery of potentially active compounds but also helps researchers deeply understand disease mechanisms at the organ level, promoting the translation of preclinical research to clinical applications.

The combination of CNN with optical microscopy and other imaging technologies has significantly improved the efficiency of analyzing cellular behaviors and morphological changes in organ-on-a-chip (Dai et al., 2023). AI-driven image recognition systems can automatically detect subtle changes after drug treatment, such as alterations in cell morphology or impaired membrane integrity, which are often important indicators of drug toxicity or efficacy, thus providing more precise information for HCS (Sabaté et al., 2023).

Typical research projects advanced by AI-integrated organ-on-a-chip systems include: the iOOC liver-kidney chip with real-time ML feedback predicted drug-induced liver injury (DILI) for 12 marketed drugs with 91% accuracy and was adopted by Pfizer for safety evaluation (Li et al., 2024); the AI-enabled heart-liver multi-organ chip accurately quantified doxorubicin-induced cardiotoxicity, guiding dose optimization and reducing adverse events in clinical trials (Deng et al., 2023); the microfluidic blood-brain barrier (BBB) chip combined with CNN imaging accelerated the discovery of small-molecule BBB penetrants for Alzheimer’s disease, with two candidates entering IND-enabling studies (Dai et al., 2023); AI-automated phenotype analysis of lung organ-chips identified baricitinib as a host-directed anti-COVID-19 agent, supporting its emergency use authorization and Phase 3 clinical trials (Joseph et al., 2022).

### 3.4 Data analysis and interpretation in HTS

Beyond image recognition, AI has become indispensable for the final steps of HTS: extracting meaningful hits from raw data, interpreting complex multi-parametric readouts, and translating screening outcomes into biological knowledge. Traditional HTS data analysis relies on fixed thresholds (e.g., Z-factor, percent inhibition) and single-endpoint readouts, which often miss subtle or synergistic effects and fail to correct for systematic batch variations. AI-driven data analysis addresses these gaps by applying ML/DL to raw or pre-processed screening data for hit identification, noise reduction, and mechanistic inference.

One major area is primary hit identification from dose-response or single-concentration screens. Deep learning models (e.g., fully connected networks, Gaussian process regression) can learn non-linear concentration-response curves and distinguish true activators from artifacts due to autofluorescence or precipitation. For instance, a deep Bayesian active learning framework has been used to prioritize hits from a 1.6-million-compound HTS campaign, achieving a 50% reduction in confirmatory screening cost while maintaining >90% hit recovery (recent advances, e.g., application of active learning in HTS). Another application is the correction of batch effects-a persistent issue in HTS-where domain-adversarial neural networks and cross-domain consistency learning (Arevalo et al., 2024) can effectively remove batch-specific noise without requiring high-quality labels, thereby improving the reproducibility of hit lists across experimental batches.

AI also enables multi-parametric analysis beyond single activity scores. In phenotypic HTS where cells are treated with thousands of compounds, DL models can integrate morphological features (from HCS), transcriptomic changes, and metabolic readouts to cluster compounds by mechanism of action. For example, the Image2Omics approach (Way et al., 2023) predicted global gene expression profiles from high-content images, allowing mechanism-of-action prediction without direct sequencing. Similarly, multi-task graph neural networks have been used to simultaneously predict compound activity against several related targets, reducing false positives caused by off-target effects (Monem et al., 2024).

Interpretability of AI models is also critical for data analysis. Explainable AI (XAI) methods such as SHAP and attention-based feature attribution are now being applied to HTS datasets to identify which molecular descriptors or image features most strongly drive hit calls. This not only helps researchers trust AI recommendations but also provides hypotheses for structure-activity relationships. For instance, in an HTS for antibacterials, an attention-enhanced GNN highlighted key chemical substructures responsible for membrane permeation, guiding subsequent medicinal chemistry (Cai et al., 2022).

Therefore, the integration of AI into HTS data analysis-from hit picking and batch correction to mechanism annotation-is no longer ancillary but essential for realizing the full potential of high-throughput technologies. For instance, in the HCS study by Lau et al. (2024), AI-based morphological profiling not only identified active anti-inflammatory compounds but also automatically classified them into known mechanism-of-action clusters, demonstrating AI’s analytical power beyond simple hit detection. In organoid screening, Fillioux et al. (2023) used spatiotemporal deep learning to extract drug response patterns from time-lapse videos, achieving a prediction error of only 0.1755-a task impossible by manual inspection.

## 4 Conclusion

The application of artificial intelligence (AI) in high-throughput screening (HTS) has greatly promoted the intelligent process of drug discovery and development. With the continuous advancement of machine learning (ML) and deep learning (DL), AI exhibits significant advantages in compound screening (including both ligand-based and structure-based virtual screening), image analysis, post-screening data analysis and interpretation, and data processing. AI not only overcomes the limitations of traditional HTS methods but also provides novel solutions for combination drug screening and multimodal data fusion. For example, graph neural network (GNN)-based compound property prediction models have successfully improved the accuracy of key indicators such as drug metabolism and toxicity prediction, offering important references for the discovery and optimization of potential active compounds and lead compounds. DL methods have also been widely used in drug-target interaction prediction, molecular generation, and high-content screening (HCS) image analysis, further accelerating the pace of drug discovery. Moreover, AI-powered data analysis pipelines now enable automatic batch effect correction, multi-parametric hit identification, and mechanism-of-action clustering, transforming raw screening data into actionable knowledge.

Although AI has been widely applied in the HTS field, some limitations still exist. In drug discovery and biomedicine, AI models need to handle high-dimensional and complex data, resulting in huge consumption of computational resources (Zhang X. Y. et al., 2023). Although DL significantly improves prediction accuracy through automatic feature extraction and nonlinear modeling, its model training requires not only a large amount of labeled data but also high computational costs (Li et al., 2022). In addition, the poor interpretability and transparency of AI models have severely restricted their progress in drug screening and bioactivity prediction. In data analysis, batch effects and data heterogeneity remain challenges, although adversarial and consistency learning methods are beginning to address them (Arevalo et al., 2024).

With the continuous improvement of computing power and the development of distributed computing technology, the training cost of AI models is expected to decrease in the future. The deep integration of cloud computing and edge computing will provide stronger technical support for large-scale data processing. In terms of model development, explainable artificial intelligence (XAI) will become a key research direction. The development of transparent and traceable algorithms will help enhance the reliability and practicality of AI in drug discovery. In the field of combination drug screening, AI will play a greater role in addressing drug resistance and the treatment of complex diseases, providing strong support for the design of innovative drug combination regimens. Furthermore, breakthroughs in multimodal learning and cross-domain data fusion will drive HTS to a higher level, enabling AI to exhibit greater application value in the deep mining and comprehensive analysis of massive data. Specifically, the emerging role of AI in HTS data analysis-from automated hit triage to mechanism prediction-will likely become a standard component of screening pipelines, further promoting personalized therapy and precision medicine. With continuous technological development and optimization, AI will be more widely used in drug discovery and disease treatment, further promoting the advancement of personalized therapy and precision medicine.
